# Novel polymorphisms and functional characterization of the prion protein gene in sparrows (*Passer montanus*)

**DOI:** 10.3389/fvets.2026.1782728

**Published:** 2026-03-11

**Authors:** Chau-Giang Truong, Da-In Choi, Byung-Hoon Jeong

**Affiliations:** 1 Korea Zoonosis Research Institute, Jeonbuk National University, Iksan, Jeonbuk, Republic of Korea; 2 Department of Bioactive Material Sciences, Jeonbuk National University, Jeonju, Jeonbuk, Republic of Korea

**Keywords:** hexapeptide tandem repeat, polymorphism, prion, *PRNP*, PrP, SNP, sparrow

## Abstract

**Background:**

Misfolding of the prion protein (PrP) into an aberrant conformation causes prion diseases in several mammalian species; however, no prion infections have been documented in birds so far. The prion protein gene (*PRNP*) has been extensively studied in mammals, but little is known about *PRNP* polymorphisms in avian species and their potential roles in resistance to prion pathogenesis. However, the genetic variation of the sparrow *PRNP* gene remains largely uncharacterized.

**Materials and methods:**

To better understand the genetic diversity of *PRNP* gene in sparrows, we sequenced the coding region from genomic DNA of 44 individuals. We analyzed the genetic characteristics of the sparrow *PRNP* gene, including genotype, allele, and haplotype distributions, as well as linkage disequilibrium (LD) among single nucleotide polymorphisms (SNPs) and insertion/deletion (indel) variants. The functional effects of the identified polymorphisms were predicted using multiple *in silico* tools, including PolyPhen-2, SIFT, AMYCO, SODA, and MutPred-Indel. In addition, the structural impact of non-synonymous substitutions was assessed by structural modeling tools, and the amino acid sequences of the hexapeptide tandem repeat were compared across avian species.

**Results:**

A total of 24 polymorphisms were identified in the sparrow *PRNP* gene, including 9 non-synonymous substitutions and 3 indels. Among these, the A121V substitution was predicted to have the most detrimental effect, causing pronounced structural perturbation and increased amyloid propensity of sparrow PrP. The L5P and W105R substitutions also showed potentially deleterious impacts on protein stability. Among the indel polymorphisms, c.190_207delAACCCGGGCTACCCCCAC and c.243_244insAACCCCGGCTACCCCCAC were predicted to reduce solubility, whereas c.225_226insAACCCGGGCTACCCCCAC increased solubility. Furthermore, sparrow PrP exhibited a comparable length to that of quail, with both species containing eight hexapeptide repeat units.

**Conclusion:**

As far as we know, this study represents the first report of PRNP genetic polymorphisms in sparrows, providing baseline data for future studies on avian prion resistance.

## 1 Introduction

Prion diseases, also known as transmissible spongiform encephalopathies (TSEs), are fatal neurodegenerative disorders that affect both humans and various mammalian species (1). The pathogenesis of prion diseases is closely linked to the abnormal conformational conversion of the prion protein (PrP), a glycosylphosphatidylinositol (GPI)-anchored cell surface glycoprotein that is abundantly expressed in neurons (2). Under certain conditions, such as exposure to exogenous prions or spontaneous genetic mutations, the normal cellular isoform (PrP^C^) can misfold into the disease-associated *β*-sheet-rich isoform (PrP^Sc^), which aggregates into amyloid plaques and exerts neurotoxic effects (3). This structural conversion underlies the pathophysiology of several prion diseases, including Creutzfeldt–Jakob disease (CJD) in humans, bovine spongiform encephalopathy (BSE) in cattle, scrapie in sheep, chronic wasting disease (CWD) in cervids, transmissible mink encephalopathy (TME) in minks, and feline spongiform encephalopathy (FSE) in cats (1, 4–8). These diseases remain untreatable, prompting continued interest in elucidating PrP biology and the mechanisms of prion pathogenesis.

To date, no naturally occurring prion disease has been reported in any avian species, contrasting sharply with the high susceptibility observed in many mammals (9). Experimental studies have shown that chickens do not develop prion-related neuropathology even when challenged with prion-infected materials, such as BSE-infected brain homogenates (10). This striking contrast in prion susceptibility between birds and mammals has driven efforts to identify avian-specific biological features, especially those related to PrP. Species-specific susceptibility or resistance to prion disease is largely determined by the molecular properties of PrP (1, 2). Even minor changes in the *PRNP* gene can significantly alter the protein’s propensity to misfold. Among genetic variants, non-synonymous single nucleotide polymorphisms (SNPs) have been most intensively studied, as they are associated with prion disease susceptibility across multiple species, such as codons 129, 200, and 219 in humans (11–13); codons 136, 154, and 171 in sheep (14–16); codons 110, 127, 142, 143, 145, 146, 211, 222, and 240 in goats (17–23); and codons 95, 96, and 132 in deer (24–28). Additionally, a 23-bp insertion/deletion (indel) in the *PRNP* promoter region has been implicated in BSE risk in cattle, particularly when combined with a 12-bp indel haplotype (29–33). In humans, octapeptide repeat insertions (OPRI) in *PRNP* have been strongly linked to the onset of prion diseases (34–37). Conversely, protective PrP variants have been identified in prion-resistant species; for instance, canine PrP contains an aspartic acid (D) at residue 159, and equine PrP carries a serine (S) at position 167 (numbered according to human PrP), both of which are hypothesized to stabilize the protein and reduce the likelihood of conversion to PrP^Sc^ (38–40). During the BSE outbreak in the United Kingdom, species such as dogs, horses, and pigs remained clinically unaffected despite consuming contaminated feed alongside cattle (41, 42). Hence, the characteristics of the *PRNP* gene play a pivotal role in species-specific genetic resistance.

Although both mammalian and avian PrPs are encoded by the *PRNP* gene, avian PrPs exhibit notable sequence differences; for example, their N-terminal repeat domain comprises hexapeptide repeats, unlike the octapeptide repeats found in mammalian PrP (43). Nevertheless, many critical functional domains of PrP, such as glycosylation sites, the hydrophobic core, and the GPI anchor region, are conserved across birds and mammals, suggesting similar biological functions (44). To better understand the differences between avian and mammalian PrP biology, our recent studies have explored the genetic diversity of *PRNP* in various bird species, which are economically important and domesticated (45–49). However, little is known about *PRNP* variation in wild birds, particularly in *Passeriformes*, which include sparrows and account for approximately 60% of all extant bird species. This lineage diverged from *Galloanserae* approximately 90 million years ago. Despite this long evolutionary history, sparrows retain several conserved genetic features, including the *PRNP* gene (50, 51). *Passer montanus* is one of the most widely distributed and ecologically representative passerine species, thereby strengthening the justification for its selection as a model within the order *Passeriformes*. Investigating *PRNP* in sparrows is therefore crucial to determine whether this small passerine species shares PrP characteristics with chickens, ducks, quails, and pheasants, thereby deepening our understanding of the evolutionary landscape of avian prion proteins.

In this study, we sequenced and analyzed the full-length *PRNP* gene from 44 individual sparrows to identify novel polymorphisms. We characterized their genotype and allele frequencies, haplotype distributions, and linkage disequilibrium (LD) patterns. We further compared the tandem repeat region of sparrow PrP with those of other bird species, evaluated the functional consequences of non-synonymous variants using *in silico* prediction tools (PolyPhen-2, AMYCO, SODA, Missense3D, SIFT, and MutPred-Indel), and modeled their potential impact on the tertiary structure of sparrow PrP.

## 2 Materials and methods

### 2.1 Sample collection

A total of 44 genomic DNA samples from sparrows (*Passer montanus*) were obtained from the National Institute of Biological Resources. These specimens were collected from multiple regions across South Korea to ensure broad geographic representation. All experimental protocols were approved by the Institutional Animal Care and Use Committee (IACUC) of Jeonbuk National University (NON2025-157-002).

### 2.2 Genetic analysis

Polymerase chain reaction (PCR) was carried out to amplify the sparrow *PRNP* gene with gene-specific primers, including *PRNP*_F as forward primer (GATGAGCACACCTCCAGTCC) and *PRNP*_R as reverse primer (TTACGGGGCGGAAAAGGAAA). These primers were designed based on the sparrow *PRNP* gene (Gene ID: 120499134) from National Center for Biotechnology Information (NCBI) (52). The PCR was performed according to the manufacturer’s protocol for the *Taq* DNA polymerase kit (BioFact, Daejeon, Korea). The PCR mixture consisted of sparrow genomic DNA (1–2 μL), a pair of designed primers (1 μL), and sterile deionized water in a total volume of 25 μL. The annealing temperature was set at 60 °C. PCR amplicons were purified using a FavorPrep gel/PCR Purification Mini Kit (FAVORGEN, Ping Tung, Taiwan) and directly sequenced with an ABI 3730xl (Applied Biosystems, Foster City, CA, United States). Finch TV software was used for genotyping analysis (Geospiza Inc., Seattle, WA, United States).

### 2.3 *In silico* analysis

Polymorphism Phenotyping v2 (PolyPhen-2), combined AMYloid and COmposition based prediction of prion-like aggregation propensity (AMYCO), Protein Solubility based on Disorder and Aggregation (SODA), Missense3D, Sorting Intolerant From Tolerant (SIFT), and MutPred-Indel were used to analyze the effect of sparrow *PRNP* polymorphisms on sparrow PrP. PolyPhen-2 is a tool that predicts the possible functional impact of SNPs on protein structure. The prediction is scored by a Naïve Bayes classifier and categorized qualitatively as “benign,” “possibly damaging” or “probably damaging” based on pairs of False Positive Rate (FPR) thresholds (53). AMYCO predicts the effects of amino acid substitutions on the aggregation properties of prion-like domains (PrLDs) using compositional analysis (via PAPA) and amyloid propensity potential (via pWALTZ). Mutations with scores below 0.45 indicate low aggregation propensity, and scores above 0.78 indicate a high risk of aggregation (54). SODA evaluates the impact of point mutations, insertions, and deletions on protein solubility by combining sequence-derived features. A positive score indicates increased solubility, while negative values suggest decreased solubility (55). Missense3D predicts the structural consequences of missense mutations based on analyzing 3D protein models and detecting structural features. It classifies mutations as “damaging” or “neutral” (56). SIFT uses sequence conservation across protein homologs to predict whether a non-synonymous mutation affects protein function. Substitutions with normalized scores ≤0.05 are considered “deleterious,” whereas those >0.05 are classified as “tolerated” (57). MutPred-Indel estimates the pathogenic potential of in-frame insertions and deletions using a machine learning model trained on sequence conservation, structural disruption, and functional impact features. It provides a probability score (0–1), where a score >0.672 indicates a likely pathogenic variant, and ≤0.672 suggests a benign effect (58).

### 2.4 Three-dimensional structure modeling and hydrogen bond analysis of amino acid substitutions in sparrow PrP

To enable comparative analysis of 3D structures among avian PrPs, structural models were generated using the SWISS-MODEL program (59). The 3D structure of chicken PrP, determined by nuclear magnetic resonance (NMR) spectroscopy, was obtained from the Protein Data Bank (PDB ID: 1U3M) (60). The 3D structure of quail PrP (codons 128–242) and wild-type sparrow PrP (codons 126–240) were predicted using SWISS-MODEL derived from the chicken PrP NMR structure (PDB ID: 1U3M.1, chain A). Model generation and selection were based on three criteria: Global Model Quality Estimation (GMQE), Qualitative Model Energy Analysis (QMEAN), and sequence identity.

AlphaFold, a deep learning-based protein structure prediction tool developed by DeepMind, was chosen to predict the 3D structure of prion protein in sparrows. The confidence of the predicted structures was evaluated by pLDDT score ranging from 0 to 100 (61). For comparative structural analysis and hydrogen bond visualization, the resulting PDB files of the sparrow PrP protein were imported into SWISS-PdbViewer application (version 4.1). The differences in hydrogen bonding patterns were recorded to analyze the potential structural impacts of missense mutations (62).

### 2.5 Phylogenetic analysis and multiple sequence alignment

A phylogenetic tree was constructed to analyze the relationship among five avian species, including chicken (NC_052553.1), pheasant (NW_022205817.1), Pekin duck (NC_092609.1), quail (NC_029537.1), and sparrow (this study) using Molecular Evolutionary Genetic Analysis X (MEGA-X) program. Branch lengths indicate the distance of evolution measured by amino acid or nucleotide substitution rates between them (63). Clustal Omega was used to align the multiple sequences of those species mentioned above to identify sparrow-specific amino acid residues in PrP (64).

### 2.6 Statistical analysis

Hardy–Weinberg equilibrium (HWE) of genotype frequencies was calculated by using Chi-square test (65, 66). Linkage disequilibrium (LD) and haplotype analyses were performed by Haploview version 4.2 (Broad Institute, Cambridge, MA, United States) (67).

## 3 Results

### 3.1 Description of novel genetic variations in the sparrow *PRNP* gene

**Figure 1:**
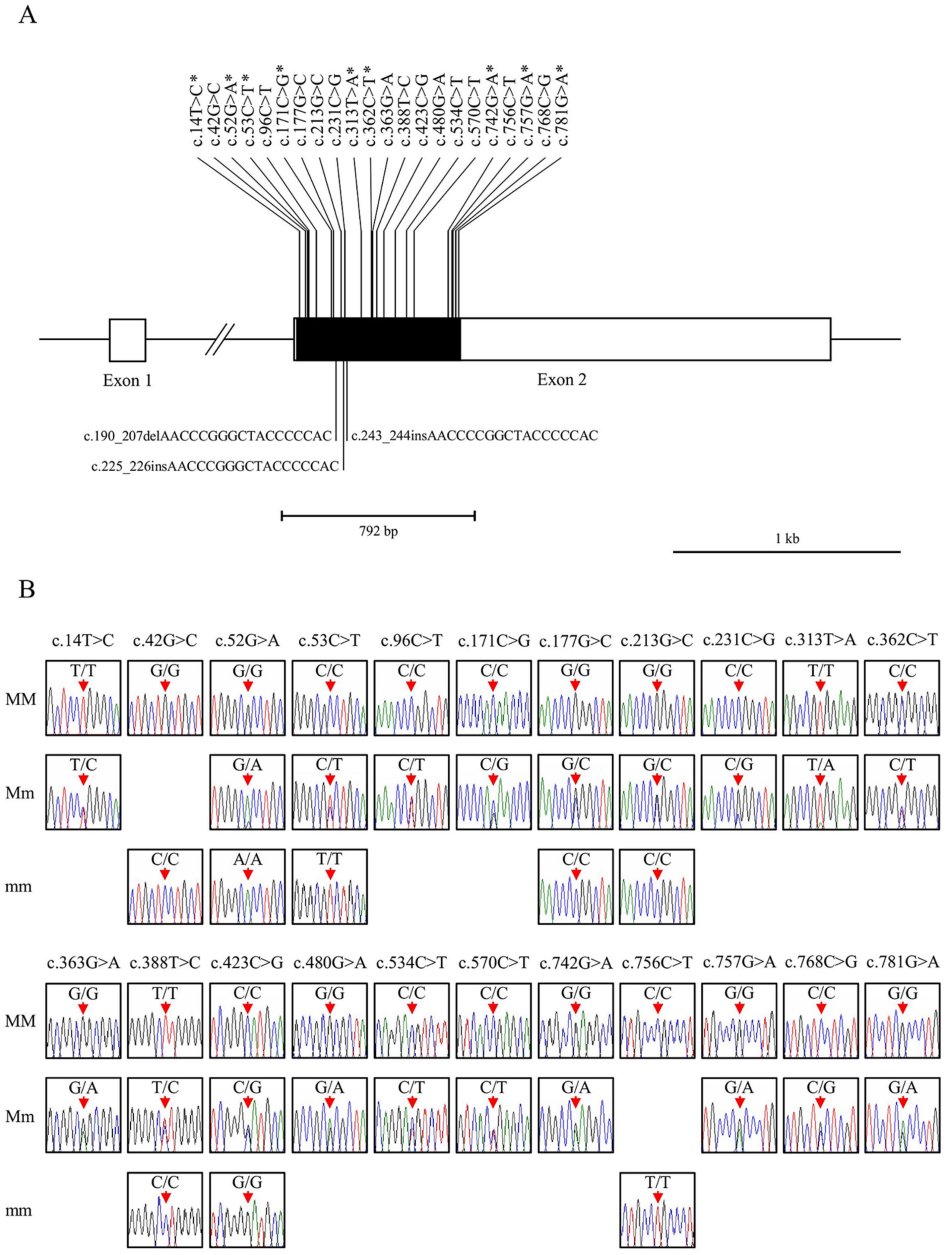
Identification of single nucleotide polymorphisms (SNPs) and insertion/deletion variants (indels) in the sparrow prion protein gene (*PRNP*). **(A)** The schematic diagram illustrates the genomic structure of the sparrow *PRNP* gene. The open reading frame (ORF) located in exon 2 is represented by a black box, while white boxes indicate the 5′ and 3′ untranslated regions (UTRs). The edged horizontal bar represents the sequenced region. The upper and lower lines display the novel SNPs and indel polymorphisms, respectively. Asterisks denote non-synonymous SNPs. **(B)** The electropherograms display two or three genotypes for the 22 novel SNPs found within the ORF of the sparrow *PRNP*. Each base in the DNA sequence is defined by the color of the peak (adenine as green, thymine as red, cytosine as blue, guanine as black). Red arrows indicate the positions of the genetic polymorphisms discovered in this study.

**Figure 2:**
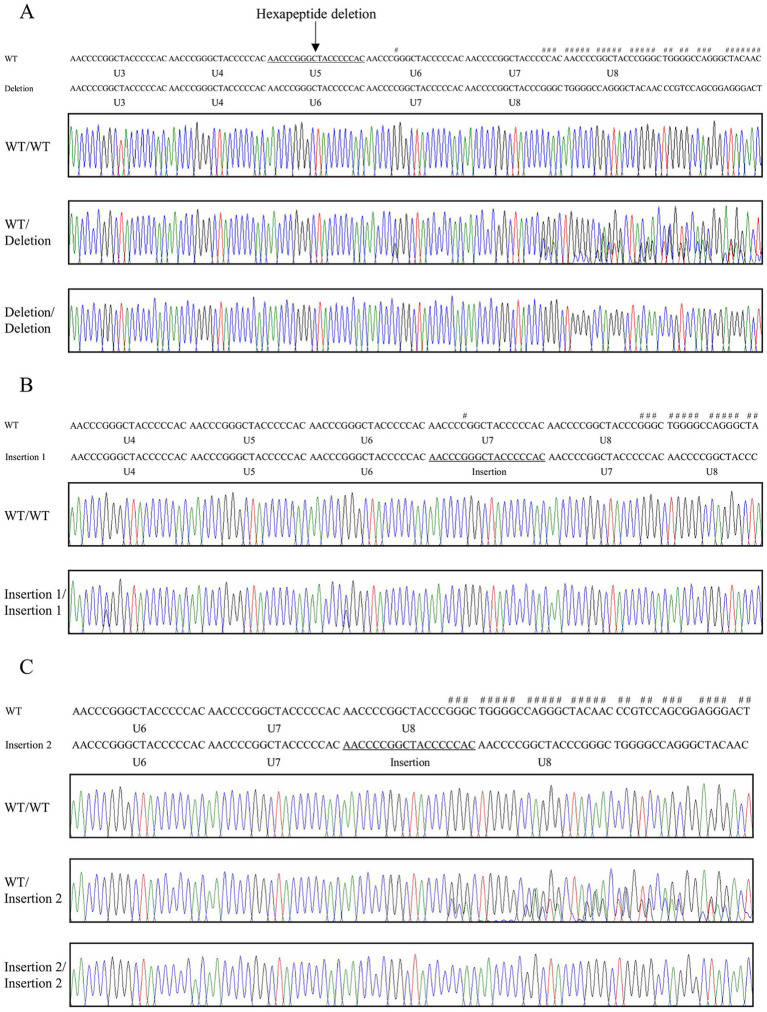
The electropherograms of the insertion/deletion polymorphisms (indels) of the prion protein gene (*PRNP*) in sparrows. **(A)** Electropherogram of c.190_207delAACCCGGGCTACCCCCAC (p.64_69NPGYPH). **(B)** Electropherogram of c.225_226insAACCCGGGCTACCCCCAC (p.75_76NPGYPH). **(C)** Electropherogram of c.243_244insAACCCCGGCTACCCCCAC (p.81_82NPGYPH). Each of the individual bases is displayed in four colors (blue: cytosine, red: thymine, black: guanine, green: adenine). Sharps (#) indicate double peaks induced by insertions/deletions in the tandem hexapeptide region. The arrow indicates the hexapeptide deletion (U5) discovered in this study. WT: the wild-type of the sparrow *PRNP* gene.

The *PRNP* gene in sparrows comprises two exons, with the open reading frame (ORF) located on exon 2 (Figure 1A). A specific primer pair was designed based on the sparrow *PRNP* gene sequence retrieved from NCBI (Gene ID: 120499134). We amplified the coding region from the genomic DNA of 44 individual sparrows and screened for genetic polymorphisms. We identified 22 novel SNPs and 3 indels within the ORF of the sparrow *PRNP* gene (Figure 1A). Interestingly, nine of these were non-synonymous substitutions that led to amino acid changes: c.14 T > C (L5P), c.52G > A (A18T), c.53C > T (A18V), c.171C > G (H57Q), c.313 T > A (W105R), c.362C > T (A121V), c.742G > A (E248K), c.757G > A (A253T), and c.781G > A (A261T). Electropherograms exhibiting the 22 SNPs represent the first report of polymorphisms in the *PRNP* gene of sparrows (Figure 1B). Furthermore, 3 indel mutations involved the increase or decrease of a hexapeptide repeat unit (NPGYPH) within the tandem repeat region designated U1 to U8. One deletion (c.190_207delAACCCGGGCTACCCCCAC) removed a single hexapeptide unit (p.64_69NPGYPH) at U5 (Figure 2A). Two insertions (c.225_226insAACCCGGGCTACCCCCAC and c.243_244insAACCCCGGCTACCCCCAC) each added one repeat unit at the positions corresponding to U7 and U8, respectively (Figures 2B,C). Heterozygotes for c.225_226insAACCCGGGCTACCCCCAC were not detected (Figure 2B). Notably, both insertions were detected in sample #27 (Supplementary Figure S1).

Genotype and allele frequencies, along with Hardy–Weinberg equilibrium (HWE) analysis, are summarized in Table 1. Linkage disequilibrium (LD) analysis based on pairwise r-squared values identified seven strong LD blocks (*r*^2^ > 0.333), detailed in Supplementary Table 1. Haplotype reconstruction revealed 18 major haplotypes (frequency >2%), with the most prevalent being TGGCCCGWWtGWtCWtTCGTCGCCGCGCG (15.1%), followed by TGGCCCGWtGWtCWtTCGCGGCCGCGCG (7.6%) and TCGTCCGDelCWtCWtTCGTGGCCGTGCG (6.8%) (Supplementary Table 2).

**Table 1:** Genotype and allele frequencies of prion protein gene (*PRNP*) polymorphisms in 44 sparrows.

Polymorphisms	Genotype frequencies, *n* (%)			Allele frequencies, *n* (%)		Hardy–Weinberg equilibrium
	MM	Mm	mm	M	m	
c.14 T > C	43 (97.73)	1 (2.27)	0 (0.00)	87 (98.86)	1 (1.14)	0.9392
c.42G > C	41 (93.18)	0 (0.00)	3 (6.82)	82 (93.18)	6 (6.82)	<0.0001
c.52G > A	41 (93.18)	2 (4.55)	1 (2.27)	84 (95.45)	4 (4.55)	0.0016
c.53C > T	31 (70.45)	8 (18.18)	5 (11.36)	70 (79.55)	18 (20.45)	0.0034
c.96C > T	43 (97.73)	1 (2.27)	0 (0.00)	87 (98.86)	1 (1.14)	0.9392
c.171C > G	40 (90.91)	4 (9.09)	0 (0.00)	84 (95.45)	4 (4.55)	0.7521
c.177G > C	40 (90.91)	2 (4.55)	2 (4.55)	82 (93.18)	6 (6.82)	<0.0001
Ins/del type 1	26 (59.09)	13 (29.55)	5 (11.36)	65 (73.86)	23 (26.14)	0.1193
c.213G > C	18 (40.91)	16 (36.36)	10 (22.73)	52 (59.09)	36 (40.91)	0.1001
Ins/del type 2	43 (97.73)	0 (0.00)	1 (2.27)	86 (97.73)	2 (2.27)	<0.0001
c.231C > G	41 (93.18)	3 (6.82)	0 (0.00)	85 (96.59)	3 (3.41)	0.8149
Ins/del type 3	41 (93.18)	1 (2.27)	2 (4.55)	83 (94.32)	5 (5.68)	<0.0001
c.313 T > A	35 (79.55)	9 (20.45)	0 (0.00)	79 (89.77)	9 (10.23)	0.4498
c.362C > T	40 (90.91)	4 (9.09)	0 (0.00)	84 (95.45)	4 (4.55)	0.7521
c.363G > A	43 (97.73)	1 (2.27)	0 (0.00)	87 (98.86)	1 (1.14)	0.9392
c.388 T > C	21 (47.73)	18 (40.91)	5 (11.36)	60 (68.18)	28 (31.82)	0.7047
c.423C > G	18 (40.91)	18 (40.91)	8 (18.18)	54 (61.36)	34 (38.64)	0.3626
c.480G > A	42 (95.45)	2 (4.55)	0 (0.00)	86 (97.73)	2 (2.27)	0.8774
c.534C > T	43 (97.73)	1 (2.27)	0 (0.00)	87 (98.86)	1 (1.14)	0.9392
c.570C > T	43 (97.73)	1 (2.27)	0 (0.00)	87 (98.86)	1 (1.14)	0.9392
c.742G > A	42 (95.45)	2 (4.55)	0 (0.00)	86 (97.73)	2 (2.27)	0.8774
c.756C > T	41 (93.18)	0 (0.00)	3 (6.82)	82 (93.18)	6 (6.82)	<0.0001
c.757G > A	43 (97.73)	1 (2.27)	0 (0.00)	87 (98.86)	1 (1.14)	0.9392
c.768C > G	41 (93.18)	3 (6.82)	0 (0.00)	85 (96.59)	3 (3.41)	0.8149
c.781G > A	39 (88.64)	5 (11.36)	0 (0.00)	83 (94.32)	5 (5.68)	0.6895

MM, Major homozygote; Mm, Heterozygote; mm, Minor homozygote; M, Major allele; m, Minor allele; Ins/del type 1, c.190_207delAACCCGGGCTACCCCCAC; Ins/del type 2, c.225_226insAACCCGGGCTACCCCCAC; Ins/del type 3, c.243_244insAACCCCGGCTACCCCCAC.

### 3.2 The computational prediction of the impacts of non-synonymous polymorphisms in the sparrow PrP

To evaluate the potential structural and functional impacts of the nine non-synonymous SNPs and three indel variants identified in the *PRNP* gene of sparrows, we performed *in silico* predictions using PolyPhen-2, SIFT, AMYCO, SODA, Missense3D, and MutPred-Indel (Table 2). PolyPhen-2 and SIFT were employed to assess the likelihood of deleterious effects caused by amino acid substitutions. Among the non-synonymous SNPs, c.362C > T (A121V) was predicted to be “possibly damaging” by PolyPhen-2 with a high confidence score of 0.997, and as “deleterious” by SIFT (0.00). Likewise, c.313 T > A (W105R) was classified as “probably damaging” (0.996) by PolyPhen-2, and c.14 T > C (L5P) was predicted to be “deleterious” by SIFT (0.00). To explore the aggregation propensity of mutant PrP proteins, we used the AMYCO tool. Of all tested variants, only codon A121V exhibited an increased aggregation tendency with a score of 0.08, suggesting a potential risk for amyloidogenic behavior. SODA was used to predict solubility changes, revealing reductions in solubility for 7 SNPs. c.53C > T (A18V), c.362C > T (A121V), and c.243_244insAACCCCGGCTACCCCCAC showed the greatest reductions in solubility relative to that of the wild-type, with solubility scores of −30.134, −21.922, and −15.06, respectively. Subsequently, structural impacts were further analyzed with Missense3D and only the A121V SNP was predicted to cause a significant structural alteration, categorized as “structural damage detected,” with a volume change of 74.088 Å^3^. For the three indel mutations, we used MutPred-Indel to evaluate their potential impact on protein structure and function. None of the indels were predicted to be deleterious based on the tool’s thresholds (Table 2).

**Table 2:** *In silico* analysis of the impact of genetic polymorphisms on the prion protein (PrP) in sparrows.

Polymorphisms	PolyPhen-2	AMYCO	SODA	Missense3D	SIFT	MutPred-Indel
c.14 T > C (L5P)	NA	Neutral (0.00)	Less soluble (−4.869)	No structural damage detected	Deleterious (0.00)	NA
c.52G > A (A18T)	NA	Neutral (0.00)	More soluble (12.423)	No structural damage detected	Tolerated (1.00)	NA
c.53C > T (A18V)	NA	Neutral (0.00)	Less soluble (−30.134)	No structural damage detected	Tolerated (0.06)	NA
c.171C > G (H57Q)	NA	Neutral (0.00)	Less soluble (−2.969)	No structural damage detected	Tolerated (0.62)	NA
c.190_207delAACCCGGGCTACCCCCAC (p.64_69NPGYPH)	NA	Neutral (0.00)	Less soluble (−15.060)	No structural damage detected	NA	Benign (0.46691)
c.225_226insAACCCGGGCTACCCCCAC (p.75_76NPGYPH)	NA	Neutral (0.00)	More soluble (12.468)	No structural damage detected	NA	Benign (0.49129)
c.243_244insAACCCCGGCTACCCCCAC (p.81_82NPGYPH)	NA	Neutral (0.00)	More soluble (12.468)	No structural damage detected	NA	Benign (0.49129)
c.313 T > A (W105R)	Probably damaging (0.996)	Neutral (0.00)	More soluble (15.255)	No structural damage detected	Tolerated (0.08)	NA
c.362C > T (A121V)	Probably damaging (0.997)	Aggregation-prone (0.08)	Less soluble (−21.922)	Structural damage detected (74.088 Å^3^)	Deleterious (0.00)	NA
c.742G > A (E248K)	NA	Neutral (0.00)	More soluble (0.041)	No structural damage detected	Tolerated (0.29)	NA
c.757G > A (A253T)	NA	Neutral (0.00)	Less soluble (−4.994)	No structural damage detected	Tolerated (0.07)	NA
c.781G > A (A261T)	NA	Neutral (0.00)	Less soluble (−5.347)	No structural damage detected	Tolerated (0.09)	NA

NA, Not available.

### 3.3 Structural effects of amino acid substitutions in the sparrow prion protein

**Figure 3:**
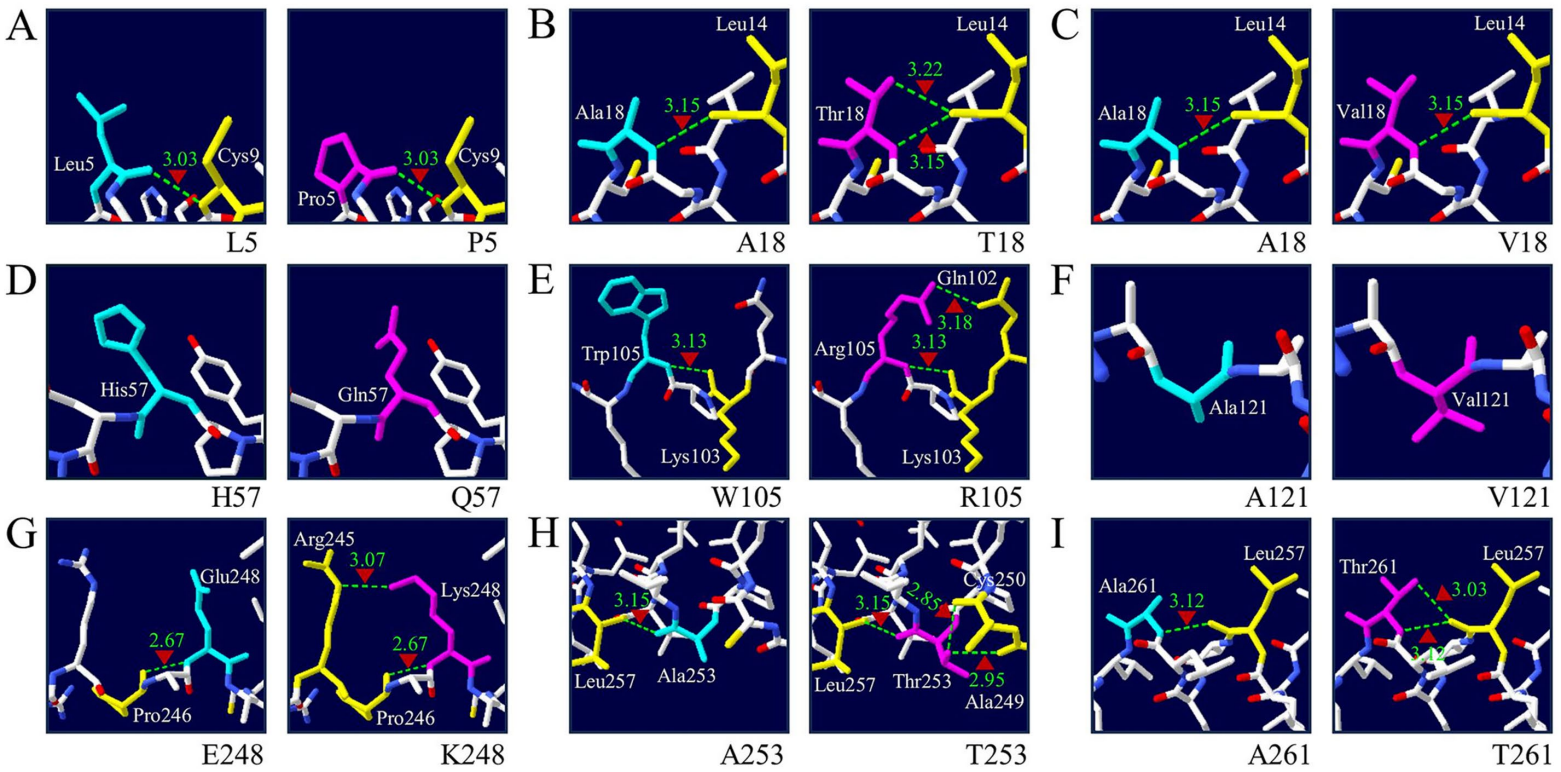
The 3D structural models and hydrogen-bonding patterns of non-synonymous single nucleotide polymorphisms (SNPs) in the sparrow prion protein (PrP). **(A)** Comparison of tertiary structures and hydrogen-bonding patterns of the sparrow PrP between L5 and P5 alleles. **(B)** Comparison of tertiary structures and hydrogen-bonding patterns of the sparrow PrP between A18 and T18 alleles. **(C)** Comparison of tertiary structures and hydrogen-bonding patterns of the sparrow PrP between A18 and V18 alleles. **(D)** Comparison of tertiary structures and hydrogen-bonding patterns of the sparrow PrP between H57 and Q57 alleles. **(E)** Comparison of tertiary structures and hydrogen-bonding patterns of the sparrow PrP between W105 and R105 alleles. **(F)** Comparison of tertiary structures and hydrogen-bonding patterns of the sparrow PrP between A121 and V121 alleles. **(G)** Comparison of tertiary structures and hydrogen-bonding patterns of the sparrow PrP between E248 and K248 alleles. **(H)** Comparison of tertiary structures and hydrogen-bonding patterns of the sparrow PrP between A253 and T253 alleles. **(I)** Comparison of tertiary structures and hydrogen-bonding patterns of the sparrow PrP between A261 and T261 alleles.

To assess the structural effect of a non-synonymous SNP on sparrow PrP, a 3D model was generated using AlphaFold2. Structural changes associated with the amino acid substitution at codon 121 were visualized using Swiss-Pdb Viewer (Supplementary Figure S2). No significant differences were observed in the overall 3D structure when compared with that of the wild-type sparrow PrP. To further analyze the potential structural impacts of the identified non-synonymous SNPs, we examined hydrogen bond (H-bond) formation in both wild-type and mutant PrP proteins using *in silico* modeling (Figure 3). First, two substitutions (H57Q and A121V) did not form any hydrogen bonds in either wild-type or mutant structures (Figures 3D,F). Second, two substitutions (L5P and A18V) retained a single H-bond similar to the wild-type (Figures 3A,C). However, four substitutions, including A18T, W105R, E248K, and A257T, introduced one additional H-bond compared to their wild-type structures (Figures 3B,E,G,I). Remarkably, the T253 allele resulted in the gain of two additional H-bonds to A249 and C250 (2.95 Å and 2.85 Å), while the A253 allele had only one H-bond with L257 (3.15 Å) (Figure 3H). The increase in H-bonds suggests that these variants may enhance local structural stability by strengthening intramolecular interactions and restricting conformational dynamics. Such alterations in the H-bond network could influence the energy landscape governing prion protein conformational conversion and aggregation propensity.

### 3.4 Analysis of evolutionary relationships and multiple sequence alignment of avian PrPs

**Figure 4:**
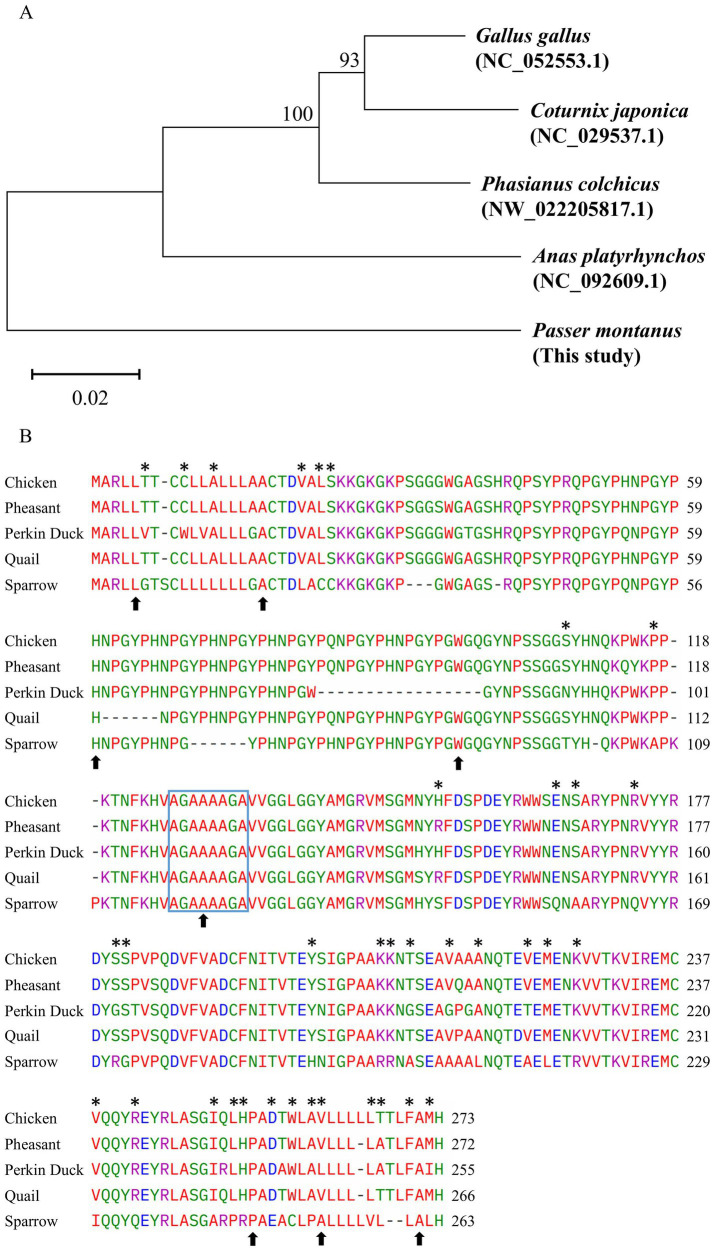
Comparison of the amino acid sequences among five avian prion proteins (PrPs). **(A)** Phylogenetic tree of the PrPs from five species. **(B)** Multiple sequence alignment of the amino acid sequences from five avian species. Colors indicate the chemical properties of amino acids (blue: acidic; red: small and hydrophobic; magenta: basic; green: hydroxyl, sulfhydryl, amine, and glycine). Asterisks indicate specific amino acids of sparrows. Arrows indicate 9 non-synonymous SNPs (L5P, A18T, A18V, H57Q, W105R, A121V, E248K, A253T, A261T). The blue box indicates the AGAAAAGA motif.

The phylogenetic analysis of PrP among five species revealed that chicken, quail, and pheasant shared a close evolutionary relationship, consistent with the conserved nature of their *PRNP* sequences. In contrast, sparrows are the most distantly related species in this phylogenetic tree (Figure 4A). Multiple sequence alignment showed that the lengths of the amino acid sequences of chicken and pheasant PrP were the longest (273 aa), whereas the shortest amino acid sequence was that of Pekin duck PrP (255 aa). The length of the amino acid sequence of sparrow PrP (263 aa) was slightly shorter than that of quail PrP (266 aa) (Figure 4B). The amino acid sequence of sparrow PrP showed the highest sequence identity with quail (79.70%), followed by chicken (79.50%), pheasant (78.97%), and Pekin duck (77.57%).

### 3.5 Comparison of tandem hexapeptide repeats in PrP of avian species

**Figure 5:**
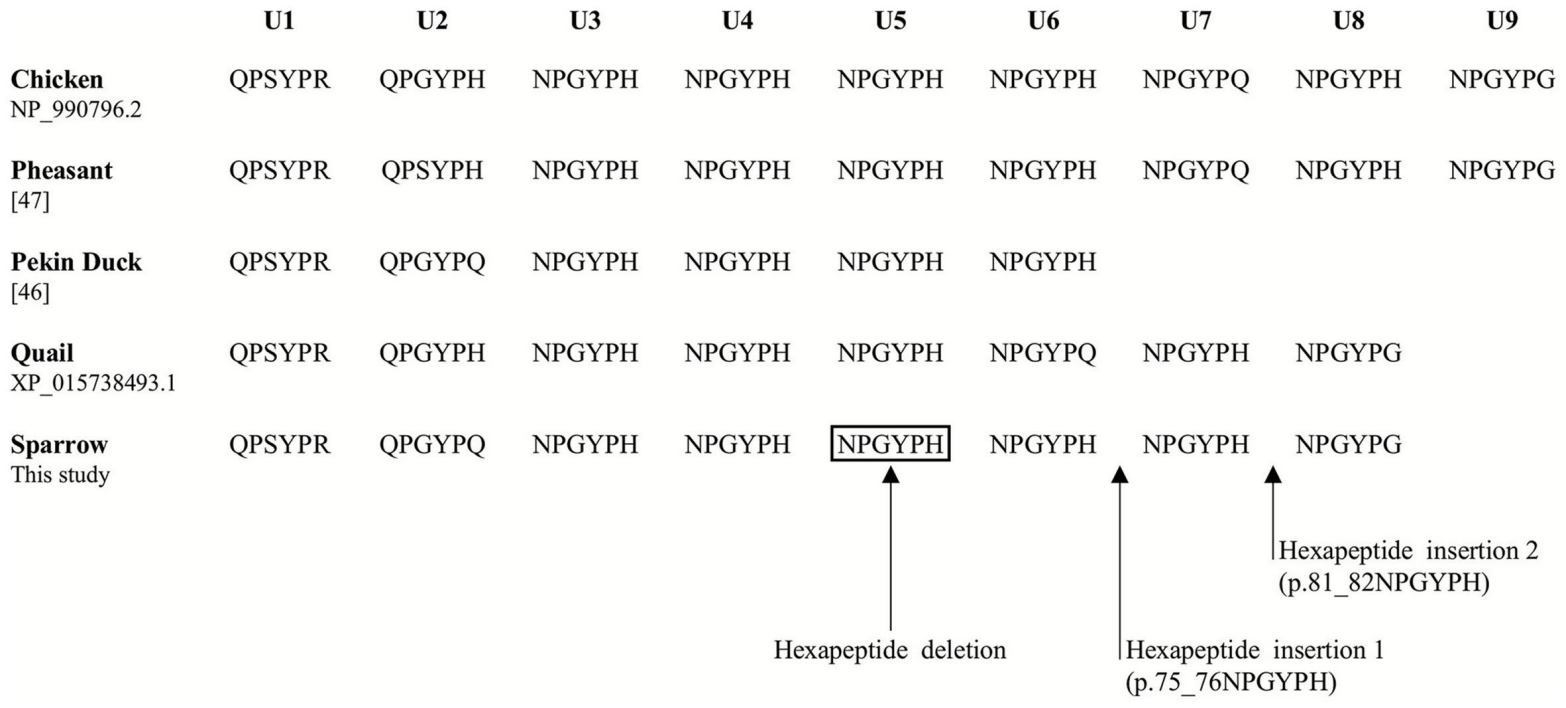
Comparison of the hexapeptide tandem repeats in several avian species. The amino acid sequences of the tandem repeat regions were retrieved from GenBank at NCBI, including chicken (NP_990796.2), pheasant (47), Pekin duck (46), quail (XP_015738493.1), and sparrow (This study). Arrows indicate the hexapeptide insertions/deletions found in the sparrow *PRNP* gene. U1–U9 indicate hexapeptide repeat units in the PrPs of five avian species.

Comparative analysis of the PrP repeat region across avian species, including chicken, Pekin duck, quail, pheasant, and sparrow, revealed that all species possess tandem hexapeptide repeats. Chicken and pheasant both possess nine repeat units, whereas quail and sparrow share the same repeat structure with 8 units. Pekin duck exhibits the shortest repeat region, comprising only six hexapeptide units. In comparison, the second and sixth units showed species-specific divergence between quail and sparrow in the conserved repeat motif (Figure 5).

### 3.6 Comparison of the secondary and tertiary structure among avian PrPs

**Figure 6:**
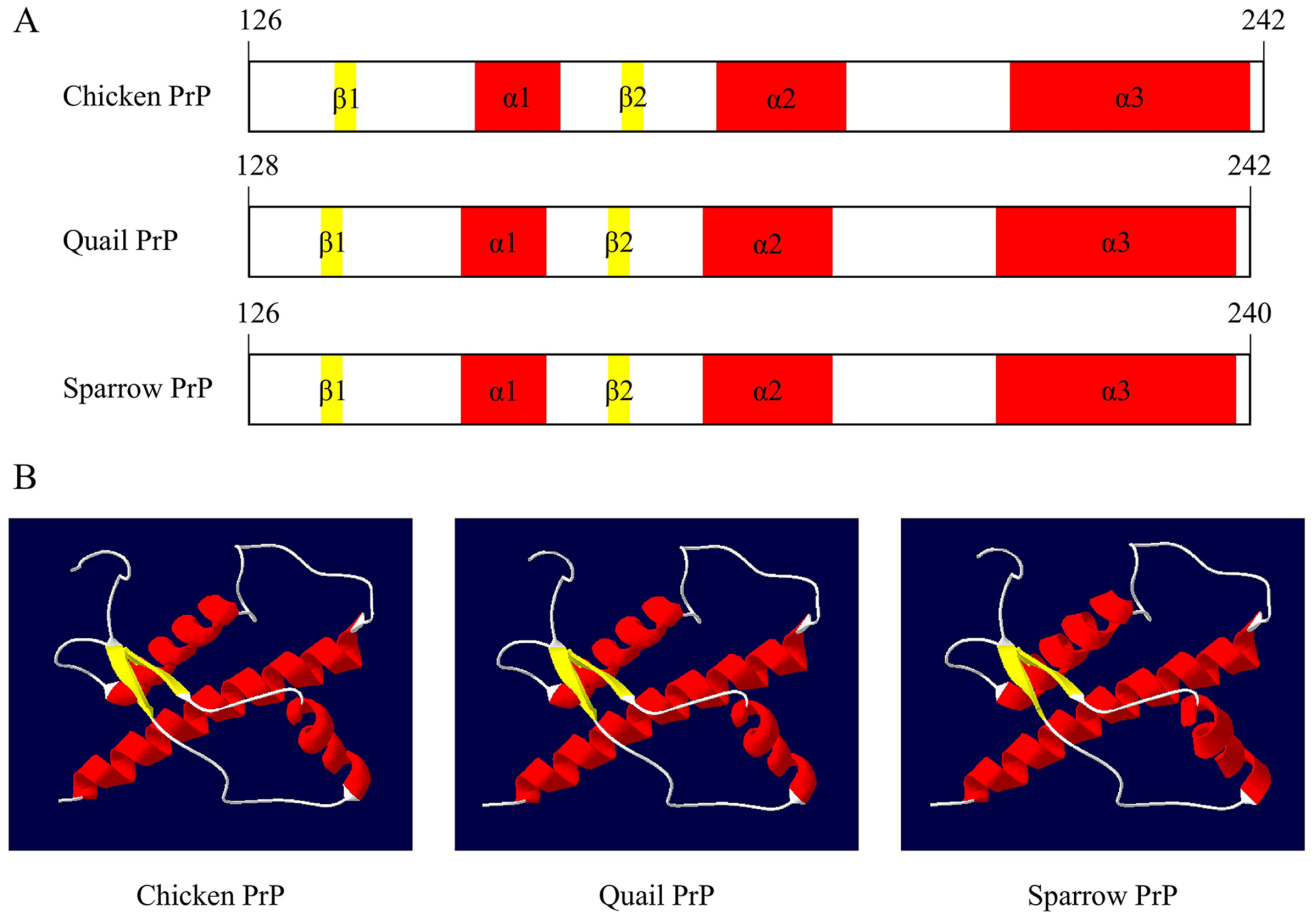
Structural comparison of avian prion proteins (PrPs). **(A)** The secondary structure composition of the avian PrPs. **(B)** The tertiary structure of the avian PrPs visualized using SWISS-Pdb Viewer. Structural elements are colored as follows: *α*-helices in red, β-sheets in yellow, and coils in white.

To compare the secondary and tertiary structures of sparrow PrP with those of other avian species, PrP models were generated using the SWISS-MODEL program and visualized with SWISS-Pdb Viewer (Figure 6; Supplementary Table S3). Sparrow PrP exhibited a secondary structure similar to that of chicken and quail PrPs, characterized by three *α*-helices and two *β*-sheets within the C-terminal globular domain (Figure 6A). The predicted 3D structures also showed a high degree of structural similarity, with no alterations in the overall PrP fold (Figure 6B). This comparative analysis indicates strong structural conservation of PrPs across avian species.

## 4 Discussion

Sparrows are one of the most prevalent avian species worldwide. To characterize the specific features of the sparrow PrP, we investigated the *PRNP* gene. In this study, we identified 25 novel polymorphisms in the sparrow *PRNP* gene, including 22 SNPs and 3 indel variants (Figures 1, 2). Such polymorphisms are of particular interest because *PRNP* variation in mammals has been closely linked to altered susceptibility to prion diseases (1, 2). Although sparrows are not known to develop prion diseases, identifying and characterizing these variants provides a valuable opportunity to explore how polymorphisms might shape PrP structure and function across species.

Among the 9 non-synonymous SNPs, A121V stood out as the most deleterious according to *in silico* analyses (Table 2). All computational tools consistently predicted that the A121V mutation could affect PrP stability and folding. This polymorphism is located at the edge of the C-terminal globular domain, which spans residues 125 to 228 in mammalian PrP and is similarly conserved in birds (44, 68, 69). This domain is vital for PrP functional properties, including structural stability, protein–protein interactions, and conversion to the pathogenic PrP^Sc^ isoform (2, 3). Moreover, the most conserved region outside the globular domain in both mammalian and avian PrPs is located between residues 113 and 137 (44). In this region, the A121 residue occurs within the highly conserved AGAAAAGA motif (Figure 3B), which spans residues 113–120 in human PrP and has been identified as a nucleation site for *β*-sheet formation during the conversion of PrP^C^ to PrP^Sc^ (70, 71). This critical motif initiates the prion misfolding process. A change from alanine to valine at this site could destabilize *β*-sheet structure and promote abnormal protein aggregation, potentially altering prion susceptibility in sparrows. This is supported by the A117V substitution, which is linked to Gerstmann-Sträussler-Scheinker disease, an inherited prion disease characterized by amyloid plaques and neurodegeneration (72, 73). Mapping onto the cryo-EM structure of CWD prion fibrils (PDB: 9DMY), residue 119 corresponding to sparrow A121 is located within a *β*-turn region connecting adjacent *β*-strands in the fibril core (74). Given the structural sensitivity of amyloid assemblies, even small side-chain differences at *β*-turn regions can influence local steric constraints and intermolecular contacts, thereby potentially modulating *β*-strand packing geometry and overall fibril stability (75). The A121V variant appears to be structurally impactful and warrants further investigation through *in vitro* or *in vivo* validation to assess its effects on PrP expression and function.

In addition, other non-synonymous SNPs also showed potential structural impacts (Table 2). The W105R SNP was predicted to damage the protein structure by PolyPhen-2, indicating that residue 105 is a potentially mutation-sensitive site in sparrow PrP. Likewise, L5P, located in the signal peptide, was predicted to be deleterious by SIFT and to significantly reduce solubility based on SODA. Therefore, the L5P SNP is likely to affect the maturation, stability, or intracellular trafficking of PrP^C^. Despite the potential for strong linkages among certain polymorphisms, our current analysis evaluated SNPs and indels individually due to the limitations of the computational tools used. Haplotype-based analysis should be considered in future investigations to provide a deeper understanding of *PRNP* variation in sparrows.

Beyond SNPs, we also observed polymorphisms in the region of tandem hexapeptide repeats located in the N-terminal domain, specifically just downstream of the signal peptide (Figure 5). In mammals, this region is analogous to the octapeptide repeat motif (PHGGGWGQ), which is known to bind copper ions (Cu^2+^), with histidine residues serving as key coordinating ligands (76, 77). Similarly, in birds, an increased number of hexapeptide repeats is hypothesized to enhance copper-binding capacity (78). Previous studies also noted that both histidine and tyrosine contribute to Cu^2+^ coordination in avian PrP (79). Remarkably, 18 of 44 sparrows (40.91%) carried either heterozygous or minor homozygous genotypes for the hexapeptide deletion (c.190_207delAACCCGGGCTACCCCCAC) (Table 1). In humans and mammals, insertion/deletion polymorphisms in the octapeptide repeat region of *PRNP* are rare and often associated with earlier disease onset and more severe symptoms (80, 81). Thus, the high frequency of these deletion alleles in sparrows suggests that they may not exert strong negative selection pressure or impair biological fitness. Conversely, hexapeptide insertions were less common, consistent with observations in other bird species such as Pekin ducks, pheasants, quails, and chickens (45, 47–49). In addition, the prion protein has been reported to undergo liquid–liquid phase separation (LLPS), a process strongly driven by its intrinsically disordered N-terminal domain. LLPS has been shown to promote *β*-sheet enrichment and protease-resistant amyloid-like assemblies, representing an early step in aggregation (82). Although LLPS alone may not generate infectious prions, it may facilitate conformational conversion under specific conditions. Therefore, insertion or deletion variants within the hexapeptide repeat region may influence LLPS propensity and modulate early conformational conversion. Further studies evaluating the copper-binding capacity and phase separation behavior with different hexapeptide genotypes of sparrow *PRNP* gene are needed to clarify their potential implications for prion-like resistance or aggregation propensity in avian PrP.

Although our *in silico* analyses suggest that specific polymorphisms, particularly A121V, may influence local structural stability and intermolecular interactions, the computational approaches have inherent limitations. Predictive models rely on algorithms and existing structural datasets, which may not fully capture protein behavior under physiological conditions. Therefore, the predicted impacts on the structural stability and functional properties of the sparrow prion protein should be interpreted with caution. Experimental validation is essential to confirm the potential misfolding propensity associated with sparrow *PRNP* polymorphisms. A subsequent step would be to assess whether these *PRNP* variants alter the kinetics of PrP misfolding or fibril formation using *in vitro* folding assays such as thioflavin T fluorescence assays, protein misfolding cyclic amplification (PMCA), or the real-time quaking-induced conversion (RT-QuIC). These approaches would enable direct comparison of aggregation propensity between wild-type and variant proteins and clarify whether the identified polymorphisms modulate conformational conversion. While these functional experiments are beyond the scope of the present genetic and computational study, they represent an important direction for future research.

It should be noted that the present study analyzed a total of 44 sparrow genomic samples. Although this sample size is relatively small for population-level genomic studies, it represents the full number of specimens currently available from the National Institute of Biological Resources. Because sparrows cannot be legally captured or euthanized for research in Korea, it was not possible to increase the number of biological replicates. Therefore, the current dataset reflects the maximum feasible sampling size. Despite this limitation, the findings provide a foundational reference for future avian *PRNP* genetic studies and highlight potential evolutionary and structural features of avian PrP.

## 5 Conclusion

In summary, our findings expand the current understanding of *PRNP* gene polymorphisms in avian species, particularly in sparrows, a species not known to develop prion diseases. The identified variants, especially A121V, may influence PrP structure, stability, and metal-binding capacity, providing insights into how similar mutations might function in mammals. Therefore, this study represents an initial step toward the hypothesis that sparrows may possess potential susceptibility to prion infection. Although these predictions require experimental validation, our results suggest that individual variants could have biological significance, contributing to genetic diversity and providing a useful comparative model for exploring PrP structure and function.

## Data Availability

The nucleotide sequence data generated in this study have been deposited in GenBank under accession number PX600118.
