# Supplementary material for: Novel polymorphisms and functional characterization of the prion protein gene in sparrows (Passer montanus)
Source: Front Vet Sci. 2026 Mar 11;13:1782728. doi: 10.3389/fvets.2026.1782728 (PMC13013064; doi:10.3389/fvets.2026.1782728)
Supplement: Supplementary file 1 [file Data_Sheet_1.docx]

Supplementary Material

# Supplementary Figures


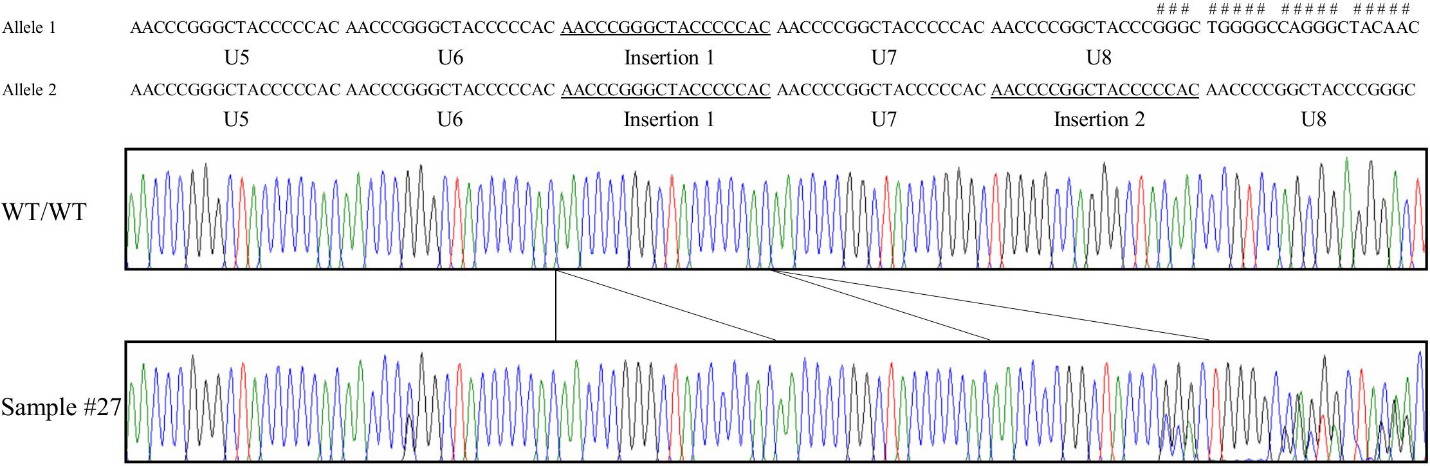


**Supplementary Figure 1.** Electropherograms of the sparrow *PRNP* gene comprising two hexapeptide insertion polymorphisms in sample #27. WT/WT: The electropherogram shows wild-type sequences without insertions at codons 75 and 81. Sample #27: The homozygous insertion sequence of c.225_226insAACCCGGGCTACCCCCAC and the heterozygous insertion sequence of c.243_244insAACCCCGGCTACCCCCAC are shown from sample #27. Insertion 1 and Insertion 2 correspond to the c.225_226insAACCCGGGCTACCCCCAC and c.243_244insAACCCCGGCTACCCCCAC polymorphisms, respectively. Four colors indicate each base of DNA sequence as follows: blue for cytosine, red for thymine, black for guanine, and green for adenine. Sharps (#) represent double peaks corresponding to hexapeptide insertions.


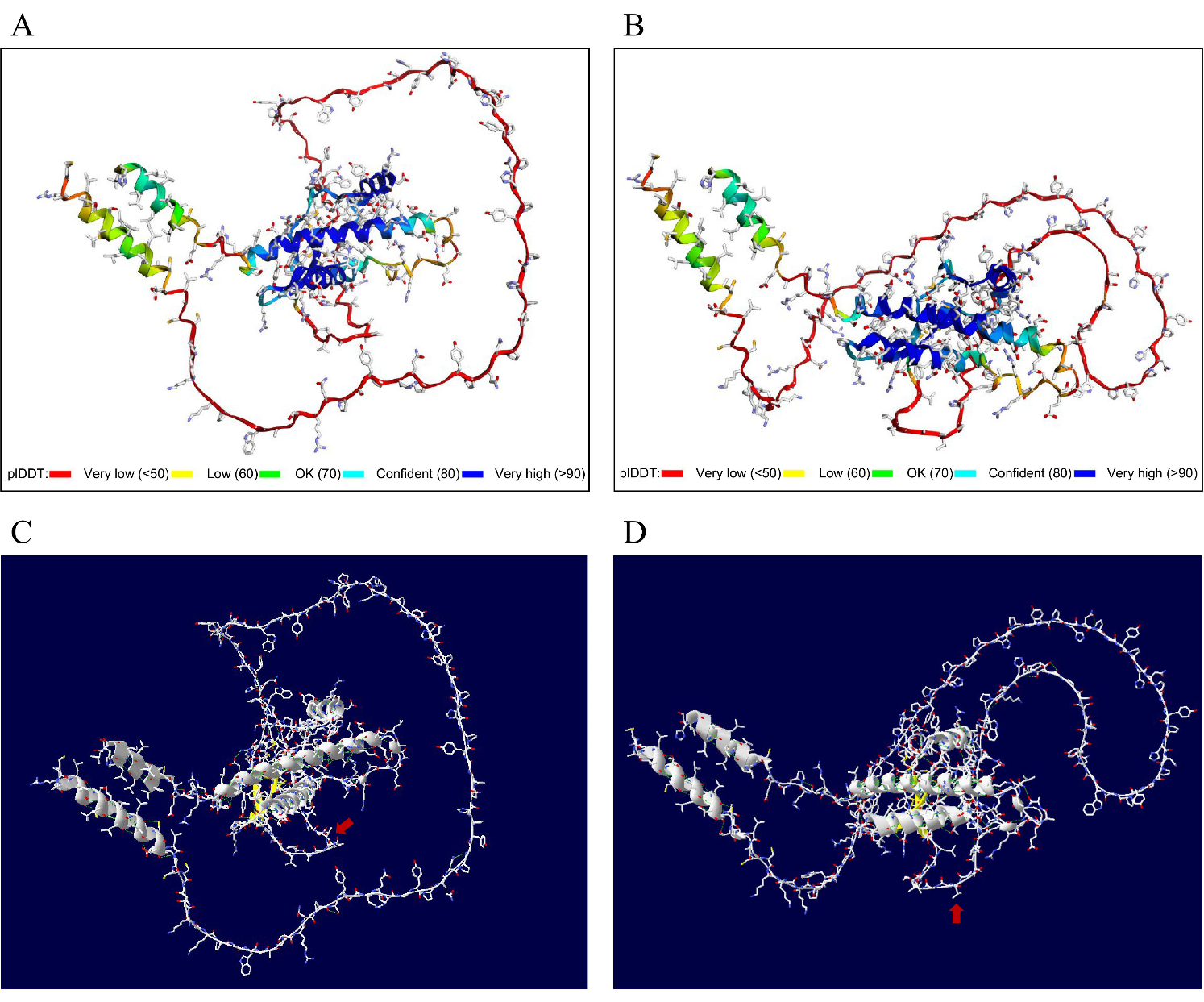


**Supplementary Figure 2.** The three-dimensional structure of prion protein in sparrow. **(A)** Tertiary structure of sparrow PrP carrying the wild-type allele, predicted using AlphaFold2. **(B)** Tertiary structure of sparrow PrP carrying the A121V polymorphism, predicted using AlphaFold2. **(C)** Hydrogen bond analysis of sparrow PrP with the wild-type allele, visualized using SWISS-Pdb Viewer. **(D)** Hydrogen bond analysis of sparrow PrP with the non-synonymous polymorphism at codon 121, visualized using SWISS-Pdb Viewer. The color gradient represents the pLDDT (predicted Local Distance Difference Test) confidence score. Hydrogen bonds are shown as green dotted lines. Helix ribbons are displayed in grey. Red arrows indicate the position of codon 121.

# Supplementary Tables

**Supplementary Table 1.** Linkage disequilibrium (LD) among genetic variations of prion protein gene (*PRNP*) in sparrows.

| ***r^2^*** | **1** | **2** | **3** | **4** | **5** | **6** | **7** | **8** | **9** | **10** | **11** | **12** | **13** | **14** | **15** | **16** | **17** | **18** | **19** | **20** | **21** | **22** | **23** | **24** | **25** |
| --- | --- | --- | --- | --- | --- | --- | --- | --- | --- | --- | --- | --- | --- | --- | --- | --- | --- | --- | --- | --- | --- | --- | --- | --- | --- |
| **1** |  |  |  |  |  |  |  |  |  |  |  |  |  |  |  |  |  |  |  |  |  |  |  |  |  |
| **2** | 0.001 |  |  |  |  |  |  |  |  |  |  |  |  |  |  |  |  |  |  |  |  |  |  |  |  |
| **3** | 0.001 | 0.003 |  |  |  |  |  |  |  |  |  |  |  |  |  |  |  |  |  |  |  |  |  |  |  |
| **4** | 0.003 | 0.285 | 0.012 |  |  |  |  |  |  |  |  |  |  |  |  |  |  |  |  |  |  |  |  |  |  |
| **5** | 0 | 0.001 | 0.001 | 0.003 |  |  |  |  |  |  |  |  |  |  |  |  |  |  |  |  |  |  |  |  |  |
| **6** | 0.001 | 0.003 | 0.002 | 0.012 | 0.001 |  |  |  |  |  |  |  |  |  |  |  |  |  |  |  |  |  |  |  |  |
| **7** | 0.001 | 0.005 | 0.003 | 0.019 | 0.001 | **0.651** |  |  |  |  |  |  |  |  |  |  |  |  |  |  |  |  |  |  |  |
| **8** | 0.004 | 0.207 | 0.017 | 0.093 | 0.004 | 0.017 | 0.026 |  |  |  |  |  |  |  |  |  |  |  |  |  |  |  |  |  |  |
| **9** | 0.008 | 0.106 | 0.033 | 0.002 | 0.017 | 0.069 | 0.006 | 0.030 |  |  |  |  |  |  |  |  |  |  |  |  |  |  |  |  |  |
| **10** | 0 | 0.002 | 0.001 | 0.006 | 0 | 0.111 | 0.068 | 0.008 | 0.001 |  |  |  |  |  |  |  |  |  |  |  |  |  |  |  |  |
| **11** | 0 | 0.003 | 0.002 | 0.009 | 0 | 0.002 | 0.003 | 0.010 | 0.024 | 0.001 |  |  |  |  |  |  |  |  |  |  |  |  |  |  |  |
| **12** | 0.001 | 0.004 | **0.427** | 0.015 | 0.001 | 0.021 | 0.005 | 0.021 | 0.042 | 0.085 | 0.002 |  |  |  |  |  |  |  |  |  |  |  |  |  |  |
| **13** | 0.001 | 0.008 | 0.002 | 0.013 | 0.001 | 0.062 | 0.020 | 0.040 | 0.079 | 0.040 | 0.009 | 0 |  |  |  |  |  |  |  |  |  |  |  |  |  |
| **14** | 0.001 | 0.003 | 0.002 | 0.005 | 0.001 | 0.002 | 0.003 | 0.017 | 0.033 | 0.001 | 0.056 | 0.003 | 0.002 |  |  |  |  |  |  |  |  |  |  |  |  |
| **15** | **1** | 0.001 | 0.001 | 0.003 | 0 | 0.001 | 0.001 | 0.004 | 0.008 | 0 | 0 | 0.001 | 0.001 | 0.001 |  |  |  |  |  |  |  |  |  |  |  |
| **16** | 0.025 | 0.034 | 0.022 | 0.120 | 0.025 | 0.012 | 0.005 | 0.078 | 0.007 | 0.011 | 0 | 0.004 | 0.053 | 0.102 | 0.025 |  |  |  |  |  |  |  |  |  |  |
| **17** | 0.018 | 0.116 | 0.030 | 0.019 | 0.007 | 0.030 | 0.046 | 0.070 | 0.082 | 0.015 | 0.004 | 0.009 | 0.072 | 0.076 | 0.018 | 0.060 |  |  |  |  |  |  |  |  |  |
| **18** | 0 | 0.002 | 0.001 | 0.006 | 0 | 0.001 | 0.002 | 0.008 | 0.034 | 0.001 | 0.001 | 0.001 | 0.003 | 0.001 | 0 | 0.011 | 0.037 |  |  |  |  |  |  |  |  |
| **19** | 0 | 0.001 | 0.001 | 0.003 | **1** | 0.001 | 0.001 | 0.004 | 0.017 | 0 | 0 | 0.001 | 0.001 | 0.001 | 0 | 0.025 | 0.007 | 0 |  |  |  |  |  |  |  |
| **20** | 0 | 0.001 | 0.001 | 0.003 | 0 | 0.001 | 0.001 | 0.004 | 0.017 | 0 | 0 | 0.001 | 0.001 | 0.241 | 0 | 0.025 | 0.018 | 0 | 0 |  |  |  |  |  |  |
| **21** | 0 | 0.002 | 0.001 | 0.006 | 0 | 0.001 | 0.002 | 0.066 | 0.034 | 0.001 | 0.001 | 0.001 | 0.003 | 0.001 | 0 | 0.05 | 0.037 | 0.001 | 0 | 0 |  |  |  |  |  |
| **22** | 0.001 | **1** | 0.003 | 0.285 | 0.001 | 0.003 | 0.005 | 0.207 | 0.106 | 0.002 | 0.003 | 0.004 | 0.008 | 0.003 | 0.001 | 0.034 | 0.116 | 0.002 | 0.001 | 0.001 | 0.002 |  |  |  |  |
| **23** | 0 | 0.001 | 0.001 | 0.045 | 0 | 0.001 | 0.001 | 0.004 | 0.008 | 0 | 0 | 0.001 | 0.001 | 0.001 | 0 | 0.005 | 0.018 | 0 | 0 | 0 | 0 | 0.001 |  |  |  |
| **24** | 0 | 0.003 | 0.002 | 0.003 | 0 | 0.002 | 0.003 | 0.012 | 0.024 | 0.001 | 0.085 | 0.002 | 0.009 | **0.741** | 0 | 0.076 | 0.056 | 0.001 | 0 | 0.326 | 0.001 | 0.003 | 0 |  |  |
| **25** | 0.001 | 0.004 | 0.003 | 0.015 | 0.001 | **0.419** | 0.263 | 0.021 | 0.087 | 0.001 | 0.002 | 0.004 | 0 | 0.003 | 0.001 | 0.129 | 0.038 | 0.001 | 0.001 | 0.001 | 0.001 | 0.004 | 0.001 | 0.002 |  |

*The value of the strong LD (r2>0.333) is emphasized in bold. 1: c.14T>C; 2: c.42G>C; 3: c.52G>A; 4: c.53C>T; 5: c.96C>T; 6: c.171C>G; 7: c.177G>C; 8: c.190_207delAACCCGGGCTACCCCCAC; 9: c.213G>C; 10: c.225_226insAACCCGGGCTACCCCCAC; 11: c.231C>G; 12: c.243_244insAACCCCGGCTACCCCCAC; 13: c.313T>A; 14: c.362C>T; 15: c.363G>A; 16: c.388T>C; 17: c.423C>G; 18: c.480G>A; 19: c.534C>T; 20: c.570C>T; 21: c.742G>A; 22: c.756C>T; 23: c.757G>A; 24: c.768C>G; 25: c.781G>A.*

**Supplementary Table 2.** Haplotype frequencies of 25 novel polymorphisms in sparrow prion protein gene (*PRNP*)*.*

|  | **c.14T>C** | **c.42G>C** | **c.52G>A** | **c.53C>T** | **c.96C>T** | **c.171C>G** | **c.177G>C** | **c.190_207delAACCCGGGCTACCCCCAC** | **c.213G>C** | **c.225_226insAACCCGGGCTACCCCCAC** | **c.231C>G** | **c.243_244insAACCCCGGCTACCCCCAC** | **c.313T>A** | **c.362C>T** | **c.363G>A** | **c.388T>C** | **c.423C>G** | **c.480G>A** | **c.534C>T** | **c.570C>T** | **c.742G>A** | **c.756C>T** | **c.757G>A** | **c.768C>G** | **c.781G>A** | **Frequency, n (%)** |
| --- | --- | --- | --- | --- | --- | --- | --- | --- | --- | --- | --- | --- | --- | --- | --- | --- | --- | --- | --- | --- | --- | --- | --- | --- | --- | --- |
| **ht1** | T | G | G | C | C | C | G | WT | G | WT | C | WT | T | C | G | T | C | G | C | C | G | C | G | C | G | 13 (15.1) |
| **ht2** | T | G | G | C | C | C | G | WT | G | WT | C | WT | T | C | G | C | G | G | C | C | G | C | G | C | G | 7 (7.6) |
| **ht3** | T | C | G | T | C | C | G | DEL | C | WT | C | WT | T | C | G | T | G | G | C | C | G | T | G | C | G | 6 (6.8) |
| **ht4** | T | G | G | C | C | C | G | WT | C | WT | C | WT | T | C | G | T | C | G | C | C | G | C | G | C | G | 6 (6.7) |
| **ht5** | T | G | G | C | C | C | G | WT | C | WT | C | WT | T | C | G | C | C | G | C | C | G | C | G | C | G | 6 (6.6) |
| **ht6** | T | G | G | C | C | C | G | DEL | G | WT | C | WT | T | C | G | T | C | G | C | C | G | C | G | C | G | 4 (4.5) |
| **ht7** | T | G | G | C | C | C | G | WT | G | WT | C | WT | A | C | G | T | G | G | C | C | G | C | G | C | G | 3 (3.5) |
| **ht8** | T | G | A | C | C | C | G | WT | G | WT | C | INS | T | C | G | T | C | G | C | C | G | C | G | C | G | 3 (3.4) |
| **ht9** | T | G | G | C | C | C | G | DEL | C | WT | C | WT | T | C | G | T | C | G | C | C | G | C | G | C | G | 3 (3.4) |
| **ht10** | T | G | G | T | C | C | G | DEL | G | WT | C | WT | A | C | G | T | C | G | C | C | G | C | G | C | G | 3 (3.3) |
| **ht11** | T | G | G | T | C | C | G | WT | G | WT | C | WT | T | C | G | C | C | G | C | C | G | C | G | C | G | 2 (2.9) |
| **ht12** | T | G | G | T | C | C | G | WT | G | WT | C | WT | T | C | G | T | C | G | C | C | G | C | G | C | G | 2 (2.8) |
| **ht13** | T | G | G | C | C | G | C | WT | C | WT | C | WT | T | C | G | C | C | G | C | C | G | C | G | C | A | 2 (2.3) |
| **ht14** | T | G | G | C | C | C | G | DEL | C | WT | C | WT | T | C | G | T | G | G | C | C | G | C | G | C | G | 2 (2.3) |
| **ht15** | T | G | G | C | C | C | C | WT | G | WT | C | WT | T | C | G | T | C | G | C | C | G | C | G | C | G | 2 (2.3) |
| **ht16** | T | G | G | C | C | C | G | DEL | G | WT | G | WT | T | C | G | T | C | G | C | C | G | C | G | C | G | 2 (2.3) |
| **ht17** | T | G | G | C | C | C | G | DEL | C | WT | C | WT | T | C | G | C | G | G | C | C | A | C | G | C | G | 2 (2.3) |
| **ht18** | T | G | G | C | C | C | G | WT | C | WT | C | WT | T | C | G | T | G | A | C | C | G | C | G | C | G | 2 (2.3) |
| **Others*** |  |  |  |  |  |  |  |  |  |  |  |  |  |  |  |  |  |  |  |  |  |  |  |  |  | 18 (20.7) |

**Others contain rare haplotypes with frequency <2.0%*

**Supplementary Table 3.** Detailed information on the secondary and three-dimensional (3D) structures of avian prion proteins (PrPs)

| **Species** | **Template** | **Range** | **Distribution of α-helices** | **Distribution of β-sheets** |
| --- | --- | --- | --- | --- |
| Chickens | 1u3m | 126-242 | 151-161, 179-194, 213-240 | 136-138, 168-170 |
| Quails | 1u3m.1.A | 128-242 | 151-161, 179-194, 213-240 | 136-138, 168-170 |
| Sparrows | 1u3m.1.A | 126-240 | 149-159, 177-192, 211-238 | 134-136, 166-168 |
